# Baseline mitral regurgitation predicts outcome in patients referred for dobutamine stress echocardiography

**DOI:** 10.1007/s10554-017-1163-6

**Published:** 2017-07-06

**Authors:** Jamie M. O’Driscoll, Paula Gargallo-Fernandez, Marco Araco, Manuel Perez-Lopez, Rajan Sharma

**Affiliations:** 1 Department of Cardiology, St George’s Healthcare NHS Trust, Blackshaw Road, Tooting, London, SW17 0QT UK; 2 School of Human and Life Sciences, Canterbury Christ Church University, Kent, UK

**Keywords:** Mitral regurgitation, Dobutamine stress, Prognosis

## Abstract

A number of parameters recorded during dobutamine stress echocardiography (DSE) are associated with worse outcome. However, the relative importance of baseline mitral regurgitation (MR) is unknown. The aim of this study was to assess the prevalence and associated implications of functional MR with long-term mortality in a large cohort of patients referred for DSE. 6745 patients (mean age 64.9 ± 12.2 years) were studied. Demographic, baseline and peak DSE data were collected. All-cause mortality was retrospectively analyzed. DSE was successfully completed in all patients with no adverse outcomes. MR was present in 1019 (15.1%) patients. During a mean follow up of 5.1 ± 1.8 years, 1642 (24.3%) patients died and MR was significantly associated with increased all-cause mortality (*p* < 0.001). With Kaplan–Meier analysis, survival was significantly worse for patients with moderate and severe MR (*p* < 0.001). With multivariate Cox regression analysis, moderate and severe MR (HR 2.78; 95% CI 2.17–3.57 and HR 3.62; 95% CI 2.89–4.53, respectively) were independently associated with all-cause mortality. The addition of MR to C statistic models significantly improved discrimination. MR is associated with all-cause mortality and adds incremental prognostic information among patients referred for DSE. The presence of MR should be taken into account when evaluating the prognostic significance of DSE results.

## Introduction

Mitral regurgitation (MR) is the most common valvular heart disease and is often clinically silent, with prevalence increasing with age. It is estimated that 5-million of the US population will be affected by moderate or severe MR by 2030 [1]. For patients with rheumatic and degenerative mitral valve disease the only definitive treatment is mitral valve repair or replacement. MR is an important long-term predictor of adverse outcome in patients with ischemic heart disease. After acute myocardial infraction, [2] coronary artery bypass graft surgery,[3] and percutaneous coronary intervention [4] outcome is related to the presence and severity of residual MR. For patients with stable coronary artery disease, MR is also associated with outcome [5].

Dobutamine stress echocardiography (DSE) is a widely accepted non-invasive test for the diagnosis, risk stratification and prognosis of coronary artery disease [6]. Several parameters have been shown to predict prognosis, such as, resting left ventricular (LV) systolic function, scar burden and the presence and extent of myocardial ischemia [7, 8]. Functional MR determined using Doppler echocardiography is an independent predictor of cardiac mortality [9]. However, the relative prognostic importance of functional MR in stable patients referred for DSE for evaluation of ischemic heart disease is less clear. This study aimed to investigate the prevalence and associated implications of functional MR with long-term mortality, collectively and independent of other echocardiographic parameters in a large cohort of patients referred for DSE.

## Method

### Study cohort

This retrospective study consisted of 6745 (3431 men and 3314 women, age 64.9 ± 12.2 years) patients from a single centre with known or suspected coronary artery disease referred for DSE in the outpatient setting between 2005 and 2012. For patients with multiple DSE studies, only the first study was considered. Clinical characteristics were recorded at the time of DSE. Exclusion criteria included patients referred for viability assessment only and those with primary mitral valve disease. This investigation conformed to the Declaration of Helsinki principles. All patients provided informed consent before testing and the local research ethics committee approved the study.

### Transthoracic echocardiography

Before DSE, all patients underwent a full cross-sectional transthoracic echocardiogram (TTE) using a General Electric Vingmed System 7. All image acquisitions and measurements were performed as recommended by the American Society of Echocardiography [10]. LV end diastolic diameter, LV end systolic diameter, interventricular and LV posterior wall thickness at end diastole were measured from parasternal M mode recordings of the LV, with the cursor at the tips of the mitral valve leaflets. LV ejection fraction was determined by the modified biplane Simpson’s rule, with measurements averaged over three cardiac cycles. The LV endocardial border was traced contiguously from one side of the mitral annulus to the other, excluding the papillary muscles and trabeculations.

Transmitral inflow was recorded using pulsed wave Doppler recordings at the mitral valve leaflet tips in the apical four-chamber view. Peak velocity of early filling (E), peak velocity of atrial filling (A), the E/A ratio and E deceleration time were measured. Pulsed wave tissue Doppler imaging was performed at the septal and lateral mitral annulus in the four-chamber view, with results averaged in order to calculate early diastolic (E’) velocities. LV filling pressure was estimated from the mitral E/E’ ratio [11].

Color flow imaging was used to determine the presence or absence of MR. In all patients with MR, the degree of MR was graded according to semi-quantitative and quantitative methods [12]. MR was then graded as none/trace, mild, moderate or severe in all patients and where available the quantified degree of MR according to RVol and EROA as previously described [13]. Two accredited TTE imaging specialists retrospectively examined all TTE data in an echocardiography core laboratory.

### Dobutamine stress echocardiography

DSE was performed according to a standard protocol [14] with dobutamine infusion starting at and increasing every 3-min with 10 µg kg^−1^ min^−1^ to a maximum of 40 µg kg^−1^ min^−1^ (stage 4). If no end-point was reached, atropine (in doses 0.25 mg up to a maximum of 2 mg) was used. Mean dobutamine dose was 33.1 ± 5 µg kg^−1^ min^−1^ and 1956 (29%) patients required atropine (1.1 ± 0.6 mg) to achieve target heart rate. Images of the heart were acquired in standard parasternal long- and short-axis and apical 2-, 3-, 4-chamber views at baseline and during stepwise infusion of dobutamine. Baseline, low-dose (heart rate 10–15 beats above baseline), peak and recovery (10-min post drug infusion) stage images were acquired as digital full cardiac cycle loops in a quad screen format and stored for off-line analysis. The LV was divided into a 17-segment model for qualitative analysis [15] and wall motion was scored on a 4-point scale (1, normal wall motion; 2, hypokinesis; 3, akinetic; and 4, dyskinetic) as is standard [14]. In patients with resting akinetic segments a biphasic response was used to indicate ischemia. Results were classified as a normal response with an overall increase in wall motion or abnormal response. An abnormal response was described as the occurrence under stress of hypokinesia, akinesia or dyskinesia in one or more resting normal segments and/or worsening of wall motion in one or more resting hypokinetic segments [16]. In this way patients were categorised as non-ischemic or ischemic. The extent and location of inducible ischemia were evaluated and a wall motion score index (WMSI) was calculated, both at rest and during stress. Non-viable myocardium was defined as resting akinetic or dyskinetic LV segment without improvement during DSE [17] and referred to as fixed wall motion abnormalities (WMA).

### Follow-up

Patients were followed up from the date of their DSE test through to December 2014 and censored at the time of death or at last known follow-up. Mortality data were established through interrogation of electronic hospital or general practitioner records, contacting patients or a family member and through the national death registry.

### Statistical analysis

Unless otherwise specified, data are presented as mean ± standard deviation or n (%). Group comparisons were performed with use of Student’s *t* test, analysis of variance, or χ^2^ test, as appropriate. The relationship between clinical characteristics, MR severity, DSE results and all-cause mortality was assessed using multivariable adjusted Cox regression analysis. The severity of MR and all-cause mortality was firstly analyzed as a categorical variable using both semi-quantitative and quantitative techniques (n = 1019) and then with the quantified degree of MR (n = 813) using both RVol and EROA. All models were adjusted for standard cardiovascular disease (CVD) risk factors and included age, gender, previous history of coronary revascularization, myocardial infarction, and presence or absence of diabetes, family history of CVD, hypercholesterolemia, hypertension, and smoking history as well as long-term cardiac medication (anti-anginal medication is defined as any treatment alone or in combination of beta-blockers, calcium antagonists, or nitrates). All other variables that reached a *p* value <0.05 were entered into the final multivariate Cox model. Hazard ratios (HR) and corresponding 95% confidence intervals (CI) are reported.

Kaplan–Meier survival curves were constructed and compared using the log-rank test and a *p* value <0.05 was used to report statistical significance. The survival curves were stratified first according to MR severity as a categorical variable, and second, according the presence or absence of MR with or without myocardial ischemia on DSE. Survival curves were then constructed according to the quantified degree of MR for both RVol (0, 1–29, and ≥30 ml) and EROA (0, 1–19, and ≥20). We then calculated the C statistic as a measure of the incremental value of selected baseline TTE parameters (LVEF, Scar, and MR) and myocardial ischemia on DSE to standard CVD risk factors (basic model) and anti-anginal therapy. All analyses were conducted using the statistical package for social sciences (SPSS 21 release version of SPSS for Windows; SPSS Inc., Chicago IL, USA).

## Results

Of 7042 patients referred for DSE between January 2005 and December 2012, 297 patients were excluded from our final analysis (104 lost at follow up, 109 referred for viability assessment only and 84 had primary mitral valve disease). The remaining 6745 patients are the subjects of this report. The patients’ mean age was 64.9 ± 12.2 years, with 51% of the population male. The prevalence of hypertension, diabetes, hypercholesterolemia, family history of CVD, and prior history of myocardial infarction and coronary revascularization were 56, 26, 46, 25, 9, 35%, respectively. 7% of patients were current smokers.

Table 1 details the baseline characteristics of the patient population according to survived and all-cause mortality. Of the demographic, clinical history, and long-term medication parameters; gender, body weight, body mass index, prevalence of hypertension, prior coronary revascularization, coronary revascularization during the follow-up period, smoking history, Canadian Cardiovascular Society (CCS) angina classification, and use of aspirin, diuretics, and anti-anginal therapy were significantly different between groups.

**Table 1:** Baseline demographic characteristics, risk factors and echocardiography measures in survived versus all-cause mortality patients

Characteristics	Survived (n = 5103)	All-cause mortality (n = 1642)	*p* value
Demographics			
Age (years)	64.8 ± 11.8	65.4 ± 13.3	0.114
Gender			<0.001
Male	2698 (52.9)	733 (44.6)	
Female	2405 (47.1)	909 (55.4)	
Height (cm)	169 ± 9.3	169 ± 9	0.926
Weight (kg)	81 ± 14.9	79.9 ± 14.4	0.009
Body mass index (kg m^2^)	28 ± 4.9	27.9 ± 5	0.009
Clinical history			
Angiogram	894 (17.5)	266 (16.2)	0.218
Hypertension	2905 (56.9)	877 (53.4)	0.034
Diabetes mellitus	1337 (26.2)	410 (25)	0.326
Hypercholesterolemia	2310 (45.3)	775 (47.2)	0.174
Family history of CVD	1240 (24.3)	425 (26.6)	0.195
Prior myocardial infarction	466 (9.1)	148 (9.3)	0.901
Prior PCI	1224 (24)	371 (23.2)	0.432
Prior CABGS	531 (10.4)	267 (16.7)	<0.001
Revascularization	515 (10.1)	195 (11.9)	0.024
Smoking history			0.001
Never smoked	3956 (77.5)	1320 (82.5)	
Ex-smoker	784 (15.4)	203 (12.7)	
Current smoker	359 (7)	115 (7.2)	
Canadian cardiovascular society angina classification			<0.001
Class I	2827 (55.4)	582 (36.4)	
Class II	1959 (38.4)	678 (42.4)	
Class III	317 (6.2)	382 (23.9)	
Long term cardiac medication			
ACE inhibitor	1863 (36.5)	580 (36.3)	0.365
Angiotensin II receptor antagonist	984 (19.3)	327 (20.4)	0.590
Aspirin	2824 (55.3)	968 (60.5)	0.012
Beta blockers	2162 (42.4)	730 (45.6)	0.148
Calcium antagonists	1582 (31)	521 (32.6)	0.602
Diuretic	1127 (22.1)	407 (25.4)	0.024
Lipid-lowering agents	3387 (66.4)	1119 (70)	0.207
Nitrates	761 (14.9)	249 (15.6)	0.819
Warfarin	322 (6.3)	100 (6.3)	0.740
At least 1 anti-anginal medication	3247 (63.6)	1108 (67.5)	0.005
Transthoracic echocardiography data			
LVESD (cm)	2.9 ± 0.7	3.2 ± 0.8	0.041
LVEDD (cm)	4.68 ± 1.2	4.92 ± 1	0.046
LV ejection fraction (%)	57.2 ± 7.7	54.7 ± 10	<0.001
Maximal LVEDD wall thickness (cm)	1.11 ± 0.3	1.18 ± 0.2	0.398
Left atrial size (mm)	36.1 ± 15	38.8 ± 14	0.06
Mitral E/A	1.08 ± 0.68	1.13 ± 0.9	0.081
Mitral E deceleration (ms)	201 ± 77	213 ± 83	0.216
Mitral E/E’	9.7 ± 5.8	11.5 ± 5.1	0.032
Mitral annular calcification	163 (3.2)	109 (6.8)	<0.001
Mitral regurgitation (MR)			<0.001
None or trace MR	4551 (89.2)	1175 (71.6)	
Mild MR	308 (6)	214 (13)	
Moderate MR	206 (4)	165 (10)	
Severe MR	38 (0.7)	88 (5.4)	
Aortic stenosis	125 (2.4)	36 (2.3)	0.556
Aortic regurgitation	115 (2.3)	55 (3.4)	0.014
Dobutamine stress echocardiography test			
Baseline heart rate (b min^−1^)	70.9 ± 15.8	68.2 ± 19.7	<0.001
Peak heart rate (b min^−1^)	135.2 ± 21.8	134.1 ± 20	0.068
Target heart rate achieved	4276 (83.8)	1384 (86.5)	0.636
Baseline sBP (mmHg)	133.3 ± 24.5	132.5 ± 24.8	0.294
Peak sBP (mmHg)	151.8 ± 38.9	151.7 ± 43.7	0.944
Baseline dBP (mmHg)	71.8 ± 20.6	72.9 ± 20.8	0.075
Peak dBP (mmHg)	74.8 ± 18.1	76.2 ± 19.2	0.011
Resting wall motion score index	1.04 ± 0.11	1.06 ± 0.14	<0.001
Peak wall motion score index	1.06 ± 0.14	1.15 ± 0.2	<0.001
Fixed wall motion abnormality	683 (13.4)	353 (22.1)	<0.001
New wall motion abnormality	929 (18.2)	713 (44.6)	<0.001

*CVD* cardiovascular disease, *PCI* percutaneous coronary intervention, *CABGS* coronary artery bypass graft surgery, *ACEI* angiotensin converting enzyme inhibitor, *LVESD* left ventricular end systolic dimension, *LVEDD* left ventricular end diastolic dimension, *LV* left ventricle, *sBP* systolic blood pressure, *dBP* diastolic blood pressure

### Transthoracic echocardiography

As shown in Table 1, left ventricular end diastolic and end systolic diameters, LVEF, mitral E/E’, and the prevalence of mitral annular calcification, MR, and aortic regurgitation were significantly different between survived versus all-cause mortality patients. MR was present in 1019 (15.1%) patients, with 522 (7.7%) graded mild, 371 (5.5%) graded moderate, and 126 (1.9%) graded severe. MR was quantitatively assessed in 813 (79.8%) of these patients, with 561 (69%) determined by the proximal isovelocity surface area technique, 191 (23.5%) by quantitative Doppler and 61 (7.5%) by both techniques. In the remaining 206 (20.2%) patients, semi-quantitative techniques were used. When the patient population is divided into groups according to MR severity using semi-quantitative and quantitative techniques (Table 2); age, the prevalence of hypertension, diabetes, hypercholesterolemia, family history of CVD, prior myocardial infarction, prior coronary revascularization, smoking history, CCS angina classification, and beta blockade use significantly differed between groups. In addition, LVEF, resting and peak WMSI, fixed and new WMA significantly differed between groups based on MR severity (Table 2).

**Table 2:** Selected characteristics of the population according to the degree of mitral regurgitation

Characteristics	Degree of mitral regurgitation				
	None/trace (n = 5726)	Mild (n = 522)	Moderate (n = 371)	Severe (n = 126)	*p* value
Demographics					
Age (years)	64.1 ± 12.3	68.5 ± 11.1	70.3 ± 9.9	72.9 ± 9.4	<0.001
Men	2901 (50.7)	273 (52.3)	198 (53.4)	59 (46.8)	0.874
History					
Angiogram	997 (17.4)	92 (17.6)	56 (15.1)	15 (11.9)	0.274
Hypertension	3282 (57.3)	261 (50)	186 (50.1)	58 (46)	<0.001
Diabetes mellitus	1518 (26.5)	114 (21.8)	92 (24.8)	23 (18.3)	0.02
Hypercholesterolemia	2661 (46.5)	205 (39.3)	158 (42.6)	61 (48.4)	0.008
Family history of CVD	1503 (26.2)	86 (16.5)	55 (14.8)	21 (16.7)	<0.001
Prior myocardial infarction	459 (8)	72 (13.8)	59 (15.9)	24 (19)	<0.001
Prior PCI	1307 (22.8)	149 (28.5)	112 (30.2)	28 (22.2)	<0.001
Prior CABGS	550 (9.6)	128 (24.5)	85 (22.9)	35 (27.8)	<0.001
Revascularization	590 (10.3)	67 (12.8)	42 (11.3)	11 (8.7)	0.268
Smoking history					<0.001
Never smoked	4403 (76.9)	457 (87.5)	318 (85.7)	98 (77.8)	
Ex-smoker	889 (15.5)	47 (9)	40 (10.8)	19 (15.1)	
Current smoker	434 (7.6)	18 (3.4)	13 (3.5)	9 (7.1)	
CCSA classification					<0.001
Class I	3103 (54.2)	165 (31.6)	112 (30.2)	29 (23)	
Class II	2161 (37.7)	245 (46.9)	173 (46.6)	58 (46)	
Class III	462 (8.1)	112 (21.5)	86 (23.2)	39 (31)	
Long term cardiac medication					
ACEI	2089 (36.5)	180 (34.5)	131 (35.3)	43 (34.1)	0.734
Angiotensin II receptor antagonist	1110 (19.4)	97 (18.6)	77 (20.8)	27 (21.4)	0.8
Aspirin	3211 (56.1)	297 (56.9)	209 (56.3)	75 (59.5)	0.885
Beta blockers	2427 (42.4)	232 (44.4)	161 (43.4)	72 (57.1)	0.01
Calcium antagonists	1779 (31.1)	166 (31.8)	112 (30.2)	46 (36.5)	0.593
Diuretic	1281 (22.4)	118 (22.6)	100 (27)	35 (27.8)	0.127
Lipid-lowering agents	3817 (66.7)	342 (65.5)	257 (69.3)	90 (71.4)	0.432
Nitrates	872 (15.2)	70 (13.4)	48 (12.9)	20 (15.9)	0.458
Warfarin	365 (6.4)	21 (4)	24 (6.5)	12 (9.5)	0.075
At least 1 anti-anginal medication	3699 (64.6)	326 (62.5)	240 (64.7)	90 (71.4)	0.306
Echocardiography parameters					
LV ejection fraction (%)	57.7 ± 7	51.2 ± 11.5	49.7 ± 12.8	49.5 ± 12.6	<0.001
Resting wall motion score index	1.03 ± 0.1	1.09 ± 0.2	1.1 ± 0.2	1.12 ± 0.2	<0.001
Peak wall motion score index	1.06 ± 0.1	1.15 ± 0.2	1.15 ± 0.2	1.2 ± 0.2	<0.001
Fixed wall motion abnormality	738 (12.9)	134 (25.7)	114 (30.7)	50 (39.7)	<0.001
New wall motion abnormality	1108 (19.4)	195 (37.4)	111 (29.9)	57 (45.2)	<0.001

### Dobutamine stress echocardiography

DSE was completed in all patients. 4457 (66.1%) patients had a normal DSE study, 1642 (24.3%) patients developed a new or worsening WMA, and 1036 (15.4%) patients had fixed WMA. Of the patients with fixed WMA, 390 (37.6%) developed a new or worsening WMA during DSE. As shown in Table 1, baseline heart rate, peak diastolic blood pressure, resting and peak WMA, and fixed and new WMA significantly differed between survived and all-cause mortality patients.

### Clinical outcomes

**Fig. 1:**
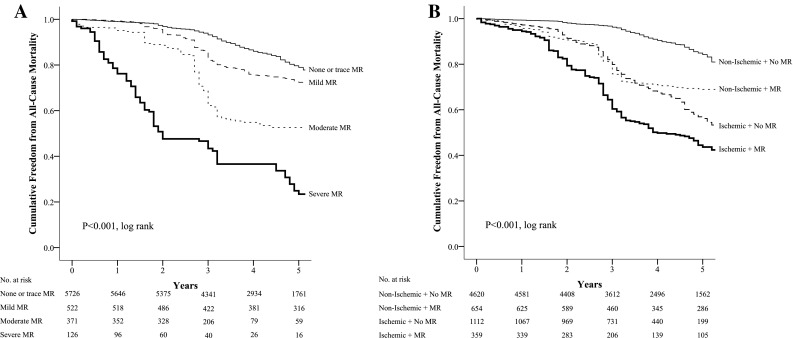
Kaplan–Meier curve for the cumulative survival and freedom from long-term mortality dichotomized according to the degree of mitral regurgitation as a categorical variable (**a**) and according to the presence or absence of mitral regurgitation with or without myocardial ischemia during dobutamine stress echocardiography (**b**)

**Fig. 2:**
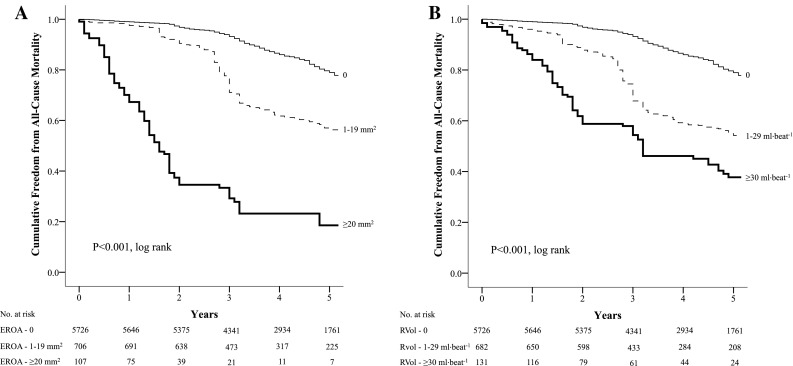
Kaplan–Meier curve for the cumulative survival and freedom from long-term mortality dichotomized according to the quantified severity of mitral regurgitation using regurgitant volume (**a**) and effective regurgitant orifice area (**b**)

During a mean follow-up period of 5.1 ± 1.8 years, all-cause mortality occurred in 1642 (24.3%) patients. The unadjusted Kaplan–Meier curves for the cumulative incidence of long-term all-cause mortality, dichotomized according to (a) the severity of MR as a categorical variable using semi-quantitative and quantitative techniques and (b) MR with or without myocardial ischemia are presented in Fig. 1. The quantified degree of MR for (a) RVol and (b) EROA are presented in Fig. 2. The differences amongst these curves were significant (all *p* < 0.001). The all-cause mortality event rate for patients with no MR was 4% per year, increasing to 8% for those with mild MR, 8.7% with moderate MR and peaking at 13.7% in those with severe MR. The all-cause mortality event rate for non-ischemic patients with no MR was 3.1% per year, increasing to 6.2% in non-ischemic patients with MR, 8.1% in ischemic patients with no MR and greatest at 13.8% in those with ischemia and MR.

Following adjusted multivariate Cox regression, the demographic, clinical history, and long-term medication parameters that independently predicted all-cause mortality were the presence of hypercholesterolemia (HR 1.25; 95% CI 1.11–1.42; *p* < 0.001), CCS angina classification Class II and Class III (HR 1.14; 95% CI 1–1.31 and HR 1.89; 95% CI 1.6–2.22; *p* < 0.001, respectively) and anti-anginal therapy (HR 1.07; 95% CI 1.03–1.19; *p* = 0.04). Previous percutaneous coronary intervention (HR 0.84; 95% CI 0.74–0.96; *p* = 0.008), coronary revascularization during the follow-up period (HR 0.67; 95% CI 0.53–0.86; *p* = 0.001) and treatment with lipid-lowering medication (HR 0.59; 95% CI 0.23–0.87; *p* = 0.02) was associated with improved survival benefit. Of the TTE parameters, moderate and severe mitral regurgitation were significantly associated with all-cause mortality (HR 2.78; 95% CI 2.17–3.57 and HR 3.62; 95% CI 2.89–4.53; *p* < 0.001, respectively), independent to parameters associated with adverse LV remodeling, including LV dimensions, LVEF, and resting and fixed WMA. When MR severity was added to the model expressed as RVol, an RVol of 1–29 ml (HR 1.99; 95% CI 1.74–2.27; *p* < 0.001) and ≥30 ml (HR 2.48; 95% CI 1.97–3.12; *p* < 0.001) were independent predictors of all-cause mortality. In addition, when MR severity was added to the model and expressed as EROA, an EROA of 1–19 mm^2^ (HR 1.84; 95% CI 1.61–2.1; *p* < 0.001) and ≥20 mm^2^ (HR 6.29; 95% CI 4.96–7.99; *p* < 0.001) were independent predictors of all-cause mortality.

DSE parameters significantly associated with all-cause mortality were resting (HR 1.07; 95% CI 1.02–1.25; *p* < 0.001) and peak WMSI (HR 17.2; 95% CI 6.43–46.15; *p* < 0.001), fixed (scar) WMA (HR 1.31; 95% CI 1.05–1.63; *p* = 0.02), and new WMA (HR 1.32; 95% CI 1.11–1.74; *p* = 0.004) (Table 3).

**Table 3:** Multivariate predictors of all-cause mortality

Characteristics		Hazard ratio (95% CI)	*p* value
Demographics			
Age (years)		1.01 (0.95–1.06)	0.82
Gender		0.95 (0.93–1.76)	0.09
Body mass index (kg·m^2^)		0.99 (0.98–1.01)	0.52
History			
Angiogram		1.24 (0.94–1.65)	0.13
Hypertension		0.39 (0.06–4.6)	0.57
Diabetes mellitus		0.74 (0.69–6.97)	0.74
Hypercholesterolemia		1.25 (1.11–1.42)	<0.001
Family history of CVD			
Prior myocardial infarction		1.07 (0.64–3.31)	0.97
Prior PCI		0.84 (0.74–0.96)	0.008
Prior CABGS		0.97 (0.83–1.13)	0.71
Revascularization		0.67 (0.53–0.86)	0.001
Smoking history			0.63
Never smoked		1 (Reference)	
Ex-smoker		1.04 (0.85–1.28)	
Current smoker		1.06 (0.89–1.26)	
Canadian Cardiovascular Society angina classification			<0.001
Class I		1 (Reference)	
Class II		1.14 (1–1.31)	
Class III		1.89 (1.6–2.22)	
Long term cardiac medication			
ACEI		0.9 (0.8–1)	0.05
Angiotensin II receptor antagonist		0.89 (0.78–1.02)	0.09
Aspirin		1.06 (0.94–1.19)	0.34
Beta blocker		1.02 (0.92–1.14)	0.69
Calcium antagonists		1 (0.9–1.11)	0.99
Diuretic		1.07 (0.95–1.21)	0.25
Lipid-lowering agents		0.59 (0.23–0.87)	0.02
Nitrates		0.95 (0.82–1.09)	0.44
Wafarin		1.06 (0.86–1.32)	0.59
At least 1 anti-anginal medication		1.07 (1.03–1.19)	0.04
Baseline echocardiography data			
LVESD (cm)		0.99 (0.87–1.14)	0.94
LVEDD (cm)		1.11 (0.9–1.36)	0.34
LV ejection fraction (%)		1 (0.99–1.01)	0.61
Mitral E/E’		0.99 (0.98–1.53)	0.32
Mitral annular calcification		1.11 (0.89–1.39)	0.34
Mitral regurgitation (MR)			<0.001
None or trace MR		1 (Reference)	
Mild MR		0.97 (0.83–1.13)	
Moderate MR		2.78 (2.17–3.57)	
Severe MR		3.62 (2.89–4.53)	
Aortic regurgitation		1.6 (0.96–1.79)	0.14
Dobutamine stress echocardiography test			
Baseline heart rate (b min^−1^)		1 (0.99–1)	0.4
Peak dBP (mmHg)		0.99 (0.99–1)	0.37
Resting wall motion score index		1.07 (1.02–1.25)	<0.001
Peak wall motion score index		17.2 (6.43–46.15)	<0.001
Fixed wall motion abnormality		1.31 (1.05–1.63)	0.02
New wall motion abnormality		1.32 (1.11–1.74)	0.004

*CVD* cardiovascular disease, *PCI* percutaneous coronary intervention, *CABGS* coronary artery bypass graft surgery, *ACEI* angiotensin converting enzyme inhibitor, *LVESD* left ventricular end systolic dimension, *LVEDD* left ventricular end diastolic dimension, *LV* left ventricle, pressure, *dBP* diastolic blood pressure

The C statistic for the basic model (CVD risk factors only) was 0.55, which failed to significantly improve with the addition of anti-anginal therapy (0.56; *p* = 0.73). However, there was a significant stepwise improvement with the addition of LVEF (0.6; *p* = 0.002), myocardial ischemia on DSE (0.6; *p* < 0.001), and MR (0.7; *p* = 0.02), which indicates an improvement in discrimination (Table 4).

**Table 4:** C statistic for the incremental value of TTE and DSE results to standard CVD risk factors

Parameter	C-statistic	*p* value
Risk factors	0.553	–
Risk factors + anti-anginal therapy	0.557	0.73
Risk factors + anti-anginal therapy + LVEF	0.595	0.002
Risk factors + anti-anginal therapy + LVEF + scar	0.602	0.27
Risk factors + anti-anginal therapy + LVEF + scar + ischemia	0.681	<0.001
Risk factors + anti-anginal therapy + LVEF + scar + ischemia + MR	0.704	0.02

*LVEF* left ventricular ejection fraction, *MR* mitral regurgitation

## Discussion

This large observational study has demonstrated that amongst patients referred for DSE, baseline MR is prevalent and a major determinant of all-cause mortality. This excess mortality was observed independently of parameters associated with LV remodeling, dobutamine induced wall motion abnormalities and traditional CVD risk factors. Similar to other ischemic MR studies [4, 13, 18–22] increasing severity of MR had a progressively negative impact on survival, with moderate and severe MR associated with poor outcome. The presence of moderate and severe MR provided incremental determination of long-term mortality when compared to established DSE markers of long-term adverse outcome, namely LV ejection fraction, scar burden, and myocardial ischemic burden. When the impact of MR severity was expressed quantitatively, a higher RVol and EROA are independently associated with greater all-cause mortality. An EROA ≥20 mm^2^ demonstrated a stronger association with all-cause mortality compared to a RVol of ≥30 ml. Nevertheless, both parameters are associated with poor outcome and allow further risk stratification of patients referred for DSE. This data supports previous research of functional MR in patients with prior myocardial infarction [13].

The AHA/ACC [23] and ESC [24] guidelines for stress echocardiography emphasize the importance of baseline LV ejection fraction, scar burden and presence and extent of myocardial ischemia as markers of adverse outcome. All of these parameters were associated with mortality in our patient population. The 5.1 ± 1.8 years mortality of this patient population was 24.3% in keeping with previous large observational DSE studies [8, 25, 26] and recently in diabetic patients referred for stress echocardiography [27]. However, the prevalence and prognostic significance of MR in a DSE population has not been specifically evaluated. MR was prevalent, being present in 15.1% of the total population and moderate and severe MR in 7.4%. As well as being independently associated with all cause mortality, the presence of moderate and severe MR provided incremental prognostic information in our population when the C statistic model was constructed using standard CVD risk factors, anti-anginal therapy, LV ejection fraction, scar, ischemic burden and MR added in the final model. We therefore believe the presence and extent of MR should be clearly documented in a DSE report and should be taken into consideration when determining the management strategy for the patient. The timing and most appropriate surgical treatment option for patients with ischemic MR remains controversial, with no convincing evidence that surgical correction improves mortality and is therefore listed as Class IIb, except when the patient is undergoing coronary artery bypass graft surgery or aortic valve repair (Class IIa) [28–30]. Current guidelines suggest patients with moderate and severe MR should have a multidisciplinary discussion involving an interventional cardiologist, mitral valve surgeon, and valve specialist [31]. In addition, correction of MR may impact other important outcomes, such as heart failure, which has significant financial implications for healthcare providers as well as patient quality of life [32–34].

Anti-anginal therapy in patients referred for stress echocardiography has been shown to adversely affect outcome [27, 35]. In our study, anti-anginal therapy was independently associated with all-cause mortality and a greater proportion of patients with severe MR were prescribed beta-blockers.

MR can be a dynamic lesion and as such its severity may change over time and under stress conditions. Evaluation of MR under exercise provides prognostic information over resting measures and identifies high-risk patients with poor outcome [36, 37]. However, DSE often improves MR [38] and patients referred are usually unable to exercise or have poor exercise capacity. This study however, has demonstrated that qualitative and quantitative baseline MR has a significant impact on all-cause mortality and should be considered in conjunction with DSE results.

### Study limitations

This is a single centre observational study and may be limited by potential referral, selection, ascertainment, and reporting biases and limited generalizability. Functional MR is a heterogeneous disease and in this study the different categories were not determined. In addition, measurements associated with MR severity are complex and subject to limitations [39]. However, quantitative techniques have been shown to be accurate and reproducible in single centers [40]. Furthermore, the dynamic nature of MR was not assessed during dobutamine infusion. However, as detailed previously, MR usually improves with dobutamine stress and the authors believe dynamic changes are better investigated using dynamic exercise.

## Conclusions

Baseline MR was independently associated with greater mortality. As such, it could be suggested that qualitative and quantitative interrogation of valvular function be routinely evaluated and reported in patients referred for DSE. The rate of all-cause mortality increased with worsening MR and was exacerbated with the addition of myocardial ischemia. In lower-risk patients optimal medical management and careful follow-up with repeated assessment of MR should be performed.
